# Utility of Chest Radiography in Emergency Department Patients Presenting with Syncope

**DOI:** 10.5811/westjem.2016.8.29897

**Published:** 2016-11-02

**Authors:** Matthew L. Wong, David Chiu, Nathan I Shapiro, Shamai A Grossman

**Affiliations:** Harvard Medical School, Beth Israel Deaconess Medical Center, Department of Emergency Medicine, Boston, Massachusetts

## Abstract

**Introduction:**

Syncope has myriad etiologies, ranging from benign to immediately life threatening. This frequently leads to over testing. Chest radiographs (CXR) are among these commonly performed tests despite their uncertain diagnostic yield. The objective is to study the distribution of normal and abnormal chest radiographs in patients presenting with syncope, stratified by those who did or did not have an adverse event at 30 days.

**Methods:**

We performed a post-hoc analysis of a prospective cohort of consecutive patients presenting to an urban tertiary care academic medical center with a chief complaint of syncope from 2003–2006. The frequency and findings for each CXR were reviewed, as well as emergency department and hospital discharge diagnoses, and 30-day outcome.

**Results:**

There were 575 total subjects, 39.7% were male, and the mean age was 57.2 (SD 24.6). Of the 575 subjects, 403 (70.1%) had CXRs performed, and 116 (20.2%) had an adverse event after their syncope. Of the 116 people who had an adverse event, 15 (12.9%) had a positive CXR, 81 (69.8%) had a normal CXR, and 20 (17.2%) did not have a CXR as part of the initial evaluation. Among the 459 people who did not have an adverse event, 3 (0.7%) had a positive CXR, 304 (66.2%) had a normal CXR, and 152 (33.1%) did not have a CXR performed. Fifteen of the 18 patients (83.4%) with an abnormal CXR had an adverse event. Eighty-one of the 385 patients (21.0%) with a normal CXR had an adverse event. Among those who had a CXR performed, an abnormal CXR was associated with increased odds of adverse event (OR: 18.77 (95% CI= [5.3–66.4])).

**Conclusion:**

Syncope patients with abnormal CXRs are likely to experience an adverse event, though the majority of CXRs performed in the work up of syncope are normal.

## INTRODUCTION

Syncope is a common symptom of what is most often a benign disease process, but it may be a marker for a life-threatening illness. Syncope accounts for 740,000 emergency department (ED) visits per year, an estimated 3% of all ED patient visits, of whom 32% get admitted to the hospital. Similarly, up to 50% of patients presenting to the ED with syncope are discharged home from the hospital without an identifying etiology.^1^–^3^ This lack of diagnostic certainty often leads to over testing. Chest radiographs are among these commonly performed tests despite their uncertain diagnostic yield.

The workup for syncope is often confused with the work up of patients with chest pain or myocardial ischemia. Yet, syncope is rarely associated with myocardial ischemia.^4^–^6^ Prior studies have shown that other tests routinely used to evaluate ischemic etiologies of syncope, such as cardiac enzyme testing in syncope, are useful only in patients with concomitant signs and symptoms of cardiac ischemia or an electrocardiogram (EKG) suggestive of a ischemic etiology.^4^–^6^ Similarly, the utility of other cardiac testing in syncope such as echocardiography may be limited to those patients with an audible murmur, a history of valvular disease, or CXR or EKG suggestive of cardiomyopathy.^6^ CXR, routinely obtained in most standard cardiac “rule out” protocols as well, has unclear utility in assessing syncope for worrisome etiologies. As such, the objective of this study is to examine the frequency of abnormal CXRs, and begin to determine if CXRs have any diagnostic value.

## METHODS

### Study Design and Setting

This is a secondary analysis of a prospective, observational, cohort study conducted in an urban teaching hospital with an annual ED census of 55,000 as part of the original Boston Syncope Criteria study. Syncope was defined as a sudden and transient (<5 minutes) loss of consciousness, producing a brief period of unresponsiveness and a loss of postural tone, ultimately resulting in spontaneous recovery requiring no resuscitation measures. More extensive details have been reported elsewhere.^6^–^7^ From September 2003 to June 2006 we studied consecutive patients presenting to the ED with syncope. Institutional review board approval was obtained prior to initiation of the study.

### Selection of Participants

Inclusion criteria included patients aged 18 years or older who met our definition of syncope.

Exclusion criteria were persistent altered mental status, alcohol- or illicit drug-related loss of consciousness, seizure, coma, hypoglycemia, transient loss of consciousness caused by head trauma, or near syncope. We excluded patients with near syncope, including all patients without transient loss of consciousness, due to a lack of consensus regarding the definition of this entity.

### Interventions

This study was observational; thus, the treating physicians were not directed to perform specific tests or work up. CXRs were ordered solely at the discretion of the treating physicians. All treatment decisions, including the necessity of a CXR, as well as the decision to admit the patient or not was at the sole discretion of the treating physician. An abnormal CXR was defined as a radiograph with findings consistent with congestive heart failure (CHF), pneumonia or pleural effusion.

### Outcome Measures

The primary outcome was the distribution of abnormal CXRs by serious adverse event. Serious adverse events were defined as death, pulmonary embolus, stroke, severe infection/sepsis, ventricular dysrhythmia, atrial dysrhythmia (including SVT [supraventricular tachycardia] and atrial fibrillation with rapid ventricular response), intracranial bleed, myocardial infarction pacemaker/implantable cardiac defibrillator placement, percutaneous coronary intervention, or surgery, blood transfusion, cardiac arrest, alteration in antidysrhythmic therapy, endoscopy with intervention, or correction of carotid stenosis. Follow up was conducted at 30 days via telephone call and medical records review. In addition to review of in-hospital and post-discharge medical records, patients were queried as to whether they had additional testing following discharge to help avoid missing results of testing done outside of our institution. Findings were considered positive if based on the discharge summary the CXR was suggestive of the etiology of the patient’s syncope or contributed to an adverse event during the patient’s care.

### Data Collection and Processing

A trained research assistant available 16 hours per day prospectively screened patients with complaints of syncope or loss of consciousness and reviewed daily patient logs to ensure completion of documentation and to identify missed off-hour patients. Patients were identified in the ED either by research assistants or by the physician caring for that patient, although the attending physician made the final decision of whether the patient met enrollment criteria. The treating physician obtained informed consent and enrolled the patient. Approximately 50% of questionnaires were completed on initial ED evaluation, with the remainder completed shortly afterward. A study investigator or trained research assistant carried out follow-up phone calls with a structured follow-up form and medical record review at 30 days after initial presentation to the ED to determine whether they had a further testing either in hospital or after discharge.

All enrolled patients had at least one episode of syncope meeting the above definition to be eligible for enrollment. All adverse outcomes or clinical interventions, such as CPR, stroke, or cardiac arrest were noted after spontaneous recovery from the initial syncopal episodes. Outcomes were determined by inpatient diagnosis, 30-day follow-up phone call, and subsequent medical records review.

### Primary Data Analysis

We queried the acquired dataset for patients who did or did not receive a CXR as part of their evaluation, as well as for whether they suffered a 30-day adverse event. Standard numerical analysis was used for reporting means and standard deviations.

## RESULTS

There were 575 people in the cohort, of whom 39.65% were male, the mean age was 57.2 (SD 24.6), and 172 (29.9%) did not have a CXR performed at all (Table 1).

Out of the 575 subjects, 403 (70.1%) had a CXR performed, and 116 (20.2%) had an adverse event after their syncope. Of the 403 people who had CXR performed, 18 (4.5%) radiographs had abnormal findings. Among the 116 people who had adverse events, 20 (17.2%) did not have a CXR done, 81 (69.8%) had a normal CXR, and 15 (12.9%) had an abnormal CXR. Among the 459 people who did not have an adverse event, 152 (33.1%) did not have a CXR performed, 304 (66.2%) had a normal CXR, and 3 (0.7%) had an abnormal CXR. In the group of 15 that had an abnormal CXR and had an adverse event, 8 (53.3%) had CHF, 4 (26.7%) had pneumonia, 2 (13.3%) had CHF as well as pneumonia, and 1 (6.7%) had an effusion. See Table 2. Further hypothesis testing using standard frequentist approaches would be difficult to interpret given the low event rate, particularly in the setting of the study’s limitations.

## DISCUSSION

The costs related to syncope-related hospital admissions total over $2 billion per year in the United States, and a large portion of these costs are directly related to diagnostic testing.^1^–^3^ Mendu and others found the yield for testing in syncope to be under 5%, with the exception of orthostatic blood pressure measurements.^8^ Whether diagnostic tests, such as chest radiographs, have a similar lack of utility among ED patients with syncope remains unclear.

Abnormal CXRs were observed in 18 of the 575 patients (3.1% overall, or 4.6% of those who had a CXR done), and 385 of the 575 patients (67.0% overall, or 95.5% of those who had a CXR done.) Patients with an abnormal CXR were much more likely to have an adverse event than not (83.4% [60.0%–95.0%] vs. 16.7% [5.0% – 40.1%]), and were at increased odds of having an adverse event compared to the group that had a normal CXR (OR [18.77], 95% CI [5.3–66.4], p<0.01) by Fisher’s exact test. All of the abnormal findings were from congestive heart failure, pneumonia, a combination of the two, or pleural effusion (Table 2). The majority of patients, however, either did not have a CXR performed (172/575, 29.9%) or had a normal CXR (385/575, 70.0%). In the subgroup of patients who ultimately had a 30-day adverse event, most CXRs were normal. The patients who did not have a CXR performed appear to be much different than the patients who did have a CXR performed, demonstrated in Table 1, which reflects discretionary physician ordering. But this research is a reflection of clinical practice; when emergency physicians elect to order CXRs, an abnormal CXR is associated with an adverse event. This suggests some modest utility in the CXR in the work up of syncope. An abnormal finding on CXR should inform clinical decision-making as those patients are likely to have an adverse event. We therefore encourage the judicious use of CXRs in the proper clinical scenario.

## LIMITATIONS

There are a number of limitations to this study. The discretionary performance of CXRs is a limitation that certainly introduces bias. Table 1 demonstrates that patients who did not have a CXR performed were much less likely to have an adverse event compared to the groups that had a normal CXR, as well as abnormal CXR. But at the same time the discretionary ordering reflects actual clinical practice. It seems unrealistic if not unethical to mandate diagnostic studies with ionization radiation for patients for whom the treating team does not think it justifiable or potentially helpful. Other limitations include the use of a single institution for a test site, which may limit the generalizability of the conclusions of this study. Furthermore, the sample size of this cohort is relatively small, as was the abnormal CXR rate, and there was a lack of long-term follow up in these patients.

## CONCLUSION

In ED patients with syncope, chest radiographs have modest diagnostic utility when ordered with discretion. Though the majority of patients who had an adverse event had a normal CXR, patients who had an abnormal CXR were at increased risk for an adverse event. When used in proper clinical context, there may be some information gained by performing a CXR in patients with syncope. A prospective study is needed to validate this conclusion. We recommend the judicious use of CXRs in the correct clinical setting.

## Figures and Tables

**Table 1:** Distribution of CXR performance and whether the patient experienced an adverse event, as well as row and columns percents, with 95% confidence intervals.

	CXR Not performed	CXR normal	CXR abnormal	
No adverse event	152	304	3	459
	(33.1% [29.0% – 37.6%])	(66.2% [61.8 – 70.4%])	(0.7% [0.13% – 2%])	
	(88.3% [82.7% – 92.4%])	(79.0% [74.5% – 83.0%])	(16.7% [5.0% – 40.1%])	
Adverse event	20	81	15	116
	(17.2% [11.4% – 25.2%])	(69.8% [61.0% – 77.5%])	(13.0% [7.9% – 20.4%])	
	(11.6% [7.6% – 17.4%])	(21.0% [17.3% – 25.4%])	(83.4% [60.0% – 95.0%])	
	172	385	18	575

*CXR,* chest radiograph.

**Table 2:** Counts of abnormal CXR findings stratified by adverse event outcome, with row and column percentages with 95% confidence intervals.

	CHF	Pneumonia	CHF & pneumonia	Effusion	
No adverse event	1	2	0	0	3
	(33.3% [5.6% – 80.0%])	(67.7% [20.2% – 94.4%])	(0% [0% – 61.8%])	(0% [0% – 61.8%])	
	(11.11% [0% – 45.7%])	(33.3% [9.3% – 70.4%])	(0% [0% – 71.0%])	(0% [0% – 83.3%])	
Adverse event	8	4	2	1	15
	(53.3% [20.1% – 75.2%])	(26.7% [10.5% – 52.4%])	(13.3% [2.5% – 39.1%])	(6.7% [0% – 32.0%])	
	(88.9% [54.3% – 100%])	(66.7% [30.0% – 90.8%])	(100% [29.0% – 100%])	(100% [16.8% – 100%])	
	9	6	2	1	18

*CXR,* chest radiograph. *CHF,* congestive heart failure.
